# Serum carcinoembryonic antigen elevation in benign lung diseases

**DOI:** 10.1038/s41598-021-98513-8

**Published:** 2021-09-24

**Authors:** Yi Yang, Mingfang Xu, Huan Huang, Xiaolin Jiang, Kan Gong, Yun Liu, Xunjie Kuang, Xueqin Yang

**Affiliations:** Cancer Department, Daping Hospital, Army Medical University, No. 10 Changjiang Zhi Road, Daping Yuzhong District, Chongqing, 400042 China

**Keywords:** Cancer, Medical research, Oncology

## Abstract

Carcinoembryonic antigen (CEA) is not only used to aid the diagnosis of lung cancer, but also help monitor recurrence and determine the prognosis of lung cancer as well as evaluate the therapeutic efficacy for lung cancer. However, studies have also shown that CEA is present at low levels in the serum of patients with benign lung diseases (BLD), which will interfere with the accurate judgment of the disease. Due to difference in sample size, detection methods, cutoff values and sources of BLD, the positive rate of CEA in BLD is different with different literature. Therefore, it is necessary to define CEA levels in patients of different BLD in a large sample study. 4796 patients with BLD were included in this study. The results showed that the CEA levels of 3.1% (149/4796) patients with BLD were elevated, with three cases exceeds 20 ng/mL (0.06%, 3/4796). The results from the literature showed that BLD had a mean positive rate of 5.99% (53/885) and only two cases had CEA above 20 ng/mL. The CEA elevations mainly distributed in chronic obstructive pulmonary disease (COPD), pneumonitis and interstitial lung disease and significantly correlated with age of patients (OR 2.69, 95% CI 1.94–3.73, p < 0.001). Pulmonary tuberculosis (7/1311, 0.53%) had the lowest positive rate of CEA elevations while pulmonary alveolar proteinosis (6/27, 22.22%) had the highest positive rate. The majority of patients with abnormally elevated CEA levels had multiple underlying diseases, mainly diseases of the circulatory system (42.28% [63/149]), endocrine diseases (26.85% [40/149]), and respiratory or heart failure (24.16% [36/149]. In endocrine diseases, 87.5% (35/40) of patients had diabetes. In conclusion, CEA is present at a low positive rate in the serum of patients with BLD, but few exceed 20 ng/mL. For lung disease patients, if CEA levels rise, we should carry out comprehensive analysis of types of lung diseases, age of patients, and comorbid diseases.

## Introduction

Carcinoembryonic antigen (CEA) was initially used to aid the diagnosis of colorectal carcinoma and other gastrointestinal tumors and monitor progression of these diseases. However, with advances in cancer diagnostics and therapy, CEA has become more widely used clinically beyond gastrointestinal tumors^1^. It is not only used to aid the diagnosis of lung cancer, but also help monitor recurrence and determine the prognosis of lung cancer as well as evaluate the therapeutic efficacy for lung cancer^2^. A prospective cohort study has demonstrated that a ≥ 14% reduction in CEA levels could predict objective response and progression-free survival (PFS), but not overall survival (OS)^3^. Recent studies have shown that CEA is closely associated with *EGFR* mutational status of lung cancer as well as with the efficacy of EGFR tyrosine kinase inhibitors (EGFR-TKI)^4,5^. In *EGFR-*mutated patients, initial CEA levels and CEA levels post progression have different prognostic values^6^.

Studies have also shown that CEA is present at low levels in the serum of patients with nonmalignant diseases and many factors affect the serum CEA levels, including physiologic, metabolic, and circulatory factors as well as detection methods^7^. A previous study^8^ showed that idiopathic pulmonary fibrosis (IPF) patients experience a rise in CEA levels. Immunohistochemical study^8^ have revealed that CEA is expressed in metaplasic bronchial epithelial cells and type II alveolar epithelial cells, indicating that apart from IPF, CEA levels may also increase in other lung diseases. With the application of immunotherapeutic agents and molecularly targeted drugs in lung cancer therapy, drug-associated lung injury and immune pneumonitis could unavoidably lead to changes in CEA levels. Komatsu et al*.*^9^ reported that CEA rose from 12.2 to 25.7 ng/mL after everolimus treatment of a patient with metastatic renal cell carcinoma; CEA declined to 5.9 ng/mL 2 months after discontinuation of everolimus. The authors believe that this was due to everolimus causing grand glass opacities in bilateral lungs. There is wide variation in the proportion of patients with benign lung diseases (BLD) with CEA elevations due to difference in sample size, detection methods, cutoff values and sources of BLD and up to 66.1% of patients with BLD reportedly have elevated CEA levels^8,10^. Therefore, it is necessary to define CEA levels in patients of different BLD. In the current study, we determined the serum CEA levels of 4,796 hospitalized patients with BLD at our tertiary care hospital.

## Patients and methods

### Patients

This retrospective study enrolled patients whose lung disease was confirmed by computed tomography (CT) scan or X-ray and who received treatment at the Department of Respiratory Medicine or Department of Thoracic Surgery at Daping Hospital, Chongqing, China between January 2007 and December 2017. The main exclusion criteria were (1) malignancy; (2) healthy subjects; (3) incomplete clinical data.

The study was approved by the Ethics Committee of Daping Hospital and conducted according to Declaration of Helsinki. Patient consent was waived by the Ethics Committee of Daping Hospital because of the retrospective nature of the study. Patient data were anonymized in this report.

### Data retrieval

We retrieved demographics including age, sex, smoking status, CEA value, and clinical data from the electronic hospital’s records system. For patients with CEA > 5 ng/mL, we obtained demographic characteristics (gender, age, and smoking status), hematological results, thyroid function, blood glucose, and liver function, radiological studies including B-ultrasound, CT, magnetic resonance imaging (MRI), and chest X-ray and medical history.

The patients with lung nodules were pathologically diagnosed by surgery, biopsy, or fiberoptic bronchoscope. If the patient showed no pathology, the medical records of one year were retrieved to confirm the disease. Malignant tumors diagnosed one year later were defined as unrelated to the tumor marker.

Serum CEA test was determined by LuminexxMAP assays and the cutoff value was set as 5 ng/mL. Nonsmoker was defined as never smoking or cessation more than 5 years.

### Data extraction from literature

We searched PubMed using the search words “CEA or carcinoembryonic antigen”, “lung” for literature published between 1995 and the date of the search. We further manually searched the references in the eligible articles for inclusion as well. Articles published in English were included. We excluded studies unrelated to BLD. Articles with no information on CEA or the maximal CEA level in BLD, or with incomplete information were also excluded. Some of the maximal CEA level was estimated from the curve images.

All data were extracted independently by two investigators (MX and IY) using a standardized form. Extracted data included the name of the first author, type of BLD, total number of patients, cutoff value of CEA, the number of patients with CEA above the cutoff value and the maximum value of CEA. Disagreements were resolved by discussion with a third investigator (HH).

### Statistical analysis

Descriptive statistics were used. All statistical analyses were performed using SPSS software package (version 16.0) (SPSS Inc., Chicago, IL, USA). Measurement data were expressed as median and range. The difference among different groups and the odds ratio were analyzed by Chi square test or one-way ANOVA. Graphs were drawn using Excel software. A p value less than 0.05 was considered statistically significant.

### Ethics approval and consent to participate

The study was approved by the Ethics Committee of Daping Hospital. *Patient consent was waived by* the Ethics Committee of Daping Hospital due to retrospective nature of study.

## Results

### Patient screening and the distribution of benign lung diseases

**Figure 1:**
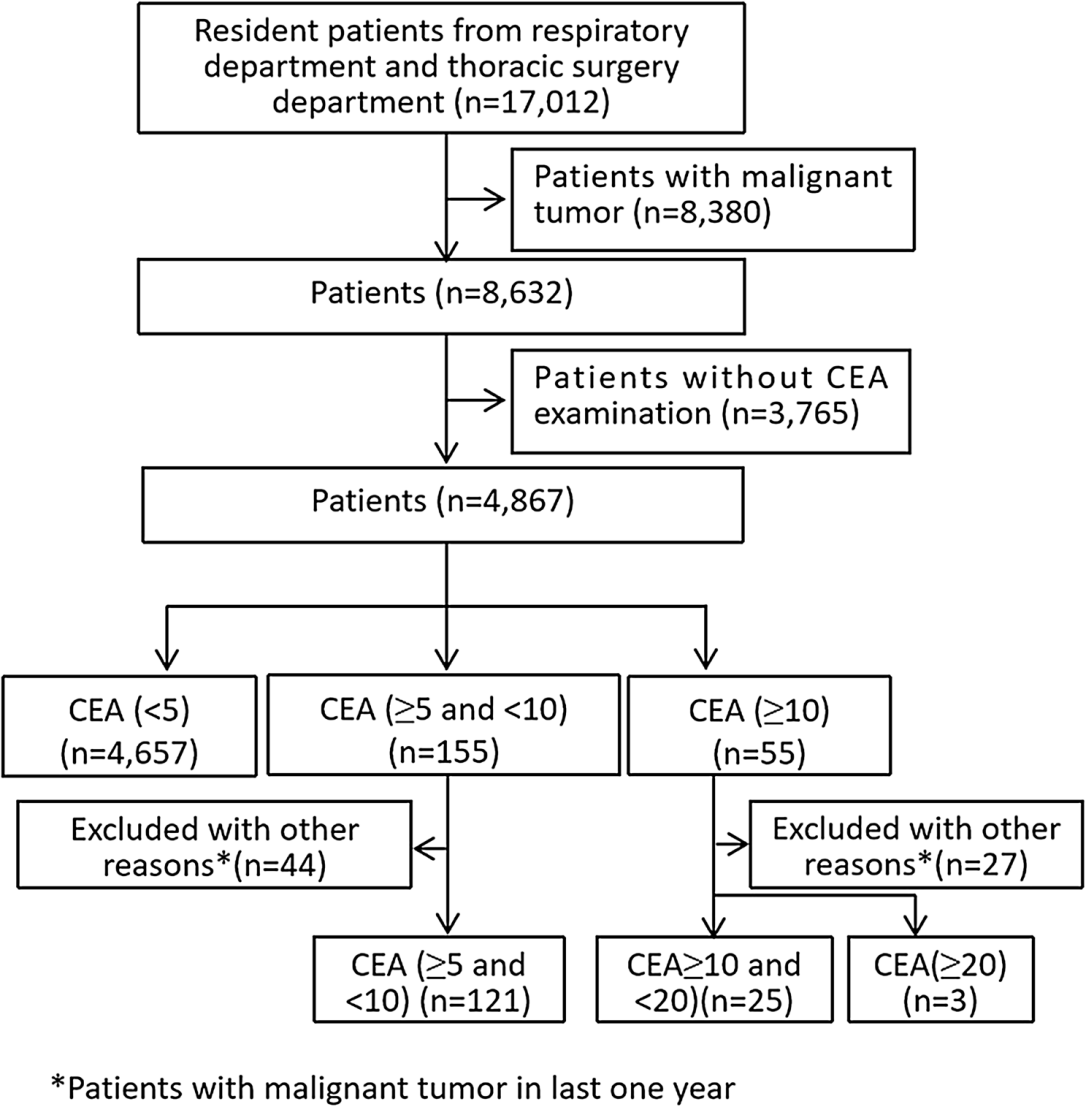
The study flowchart.

The study flow chart is shown in Fig. 1. Totally 17,012 cases received treatment between January 2007 and December 2017. We excluded 8380 cancer patients. In addition, 3765 patients with no data on CEA were excluded. Totally 4867 patients met the eligibility requirements. Seventy-one patients were diagnosed with cancer during the course of follow up and were excluded from final analysis. Finally, 4796 patients were included in the final analysis, with 3249 male patients and 1547 female patients. Their median age was 58 years (range 18, 90). Pulmonary tuberculosis was the most frequent benign lung disease (1311, 27.34%), followed by COPD (1270, 26.48%) and inflammatory pseudotumor (1018, 21.23%). The distribution of other benign diseases is shown in Table 1.

**Table 1:** Serum CEA contents according types of benign lung diseases.

Diagnose	N	N of CEA > 5 mg/mL	N of CEA > 10 mg/mL	Maximum value
COPD	1270	61 (4.80%)	12 (0.94%)	17.33
Pneumonitis/COP	484	23 (4.75%)	3 (0.62%)	40.12
IP	1018	14 (1.38%)	1 (0.09%)	11.22
Tuberculosis	1311	7 (0.53%)	1 (0.07%)	10.18
Pulmonary abscess	290	4 (1.38%)	1 (0.35%)	11.73
Bronchiectasis	110	5 (4.54%)	0	8.7
Pneumothorax	62	3 (4.83%)	0	6.61
Asthma	46	5 (10.87%)	2 (4.35%)	10.04
ILD/CTD-ILD	115	19 (16.52%)	4 (3.48%)	24.25
Pulmonary embolism	63	2 (3.17%)	1 (1.59%)	11.78
PAP	27	6 (22.22%)	3 (11.11%)	28.02
Total	4796	149 (3.11%)	28 (0.58%)	–

*COPD* Chronic obstructive pulmonary disease, *ILD* Interstitial lung disease, *CTD-ILD* Connective tissue disease-associated interstitial lung disease, *PAP* Pulmonary alveolar proteinosis, *IP* Inflammatory pseudotumor, *COP* cryptogenic organizing pneumonia.

In this study, benign lung nodules mainly included inflammatory pseudotumor (n = 1018) and tuberculoma (n = 192). Most of them were confirmed by pathology. Only 3.1% (n = 32) of inflammatory pseudotumor and 9.9% (n = 19) of tuberculoma were diagnosed by patients’ imaging, clinical symptom and laboratory examination. All the lung nodules without pathology have been confirmed by one-year follow-up.

### Patient serum CEA contents

The mean serum CEA content was 1.51 ± 1.12 ng/mL (median, 1.39; range 0.02, 40.12). CEA was > 5 ng/ mL in 149 (3.11%) patients, > 10 ng/mL in 28 (0.58%) patients and > 20 ng/mL in 3 (0.06%) patients (Table 1). More males (67.78%) had CEA > 5 ng/mL than females (32.21%). Patients with pulmonary alveolar proteinosis had the highest positive CEA rate (22.22%), followed by interstitial lung disease (ILD) [including connective tissue disease-associated interstitial lung disease (CTD-ILD)] (16.52%) and asthma (10.87%). Among benign diseases with more than 1000 cases, pulmonary tuberculosis patients had the lowest positive rate (0.53%, 7/1311) while COPD patients had the highest CEA positive rate (4.8%, 61/1270) (Table 1 and Supplementary Table 1 online).

The highest CEA level in this study was 40.12 ng/mL. This patient had cough and intermittent hemoptysis for one year, and two examinations within 2 months before surgery showed CEA values of 36.1 ng/mL and 40.12 ng/mL, respectively, with a concurrent significant rise in CA125. Two preoperative CT scans revealed obstructive inflammation of the right lower lobe. Bronchofibroscopy suggested mucosal hyperemia in bronchi of various lobes of the right lung, and the lumen became narrowed due to mucosal swelling, especially in the lower right lung. Biopsy suggested chronic mucositis. Finally, wedge resection of the lower lobe of the right lung was carried out and postoperative pathology showed cryptogenic organizing pneumonia (COP), likely due to aspiration-induced coughing a year ago. CEA levels returned to normal after surgery.

### Correlates of CEA elevations

When the cutoff value of CEA was set as 5 ng/mL, age > 65 years were 2.69 (95% CI 1.94–3.73, p < 0.001) times more likely to have CEA elevations than patients aged < 65 years (Table 3 and Supplementary data). However, gender (OR 1.00, 95% CI 0.71–1.42; p = 0.991) and smoking history (OR 1.11, 95% CI 0.80–1.54; p = 0.535) were not significant risk factors for CEA elevations (Table 2).

**Table 2:** Risk factors of CEA abnormalities.

	CEA (< 5 ng/mL)	CEA (≥ 5 ng/mL)	OR	95% CI	p
**Age (years)**					
< 65	3298	71	2.686	1.935–3.727	< 0.001
≥ 65	1349	78			
**Sex**					
Male	3148	101	1.002	0.707–1.421	0.991
Female	1499	48			
**Smoking status**					
Smoker	2064	70	1.109	0.800–1.538	0.535
Nonsmoker	2583	79			

**Figure 2:**
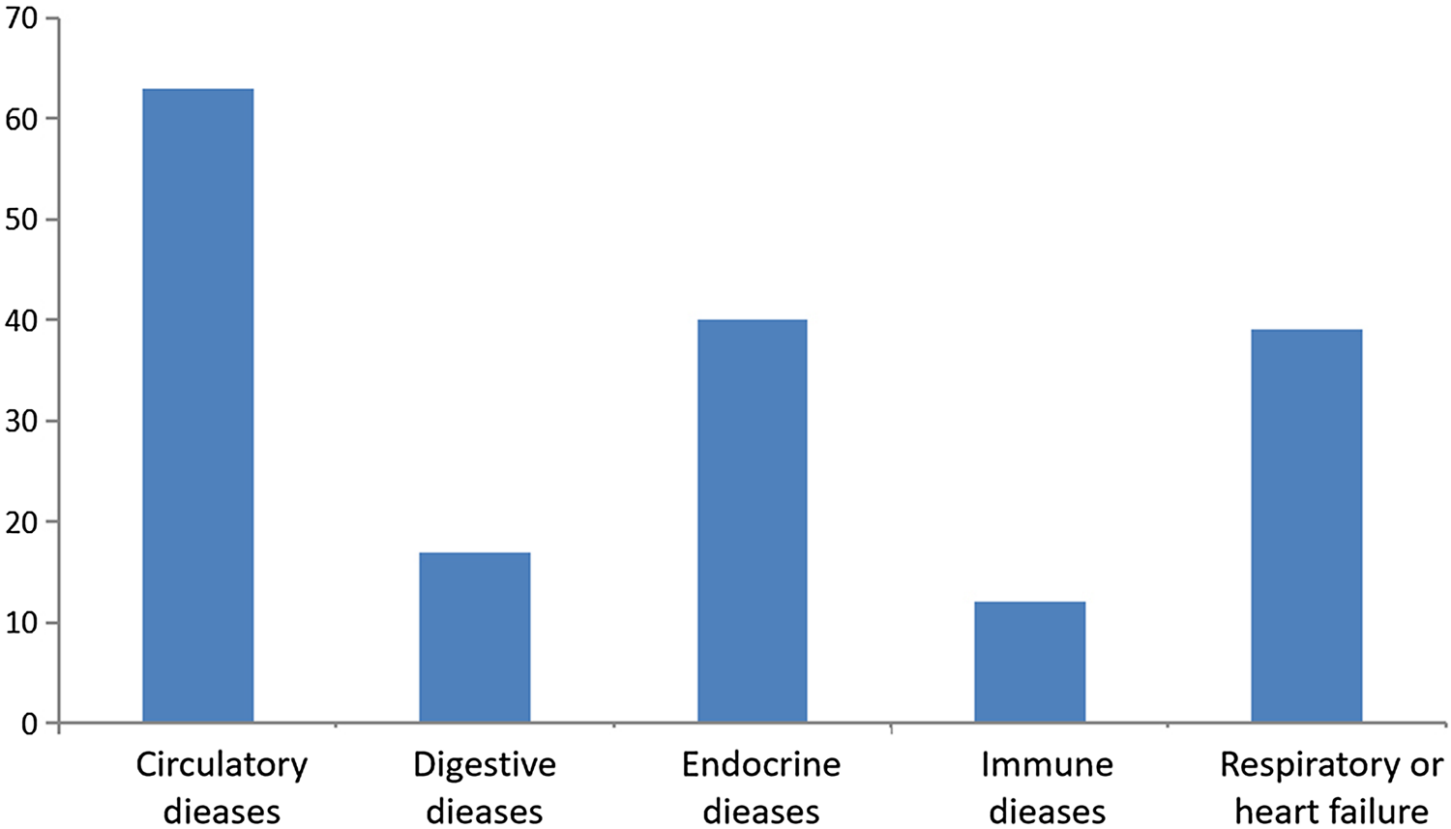
Concomitant diseases in patients with CEA levels above 5 ng/mL.

**Figure 3:**
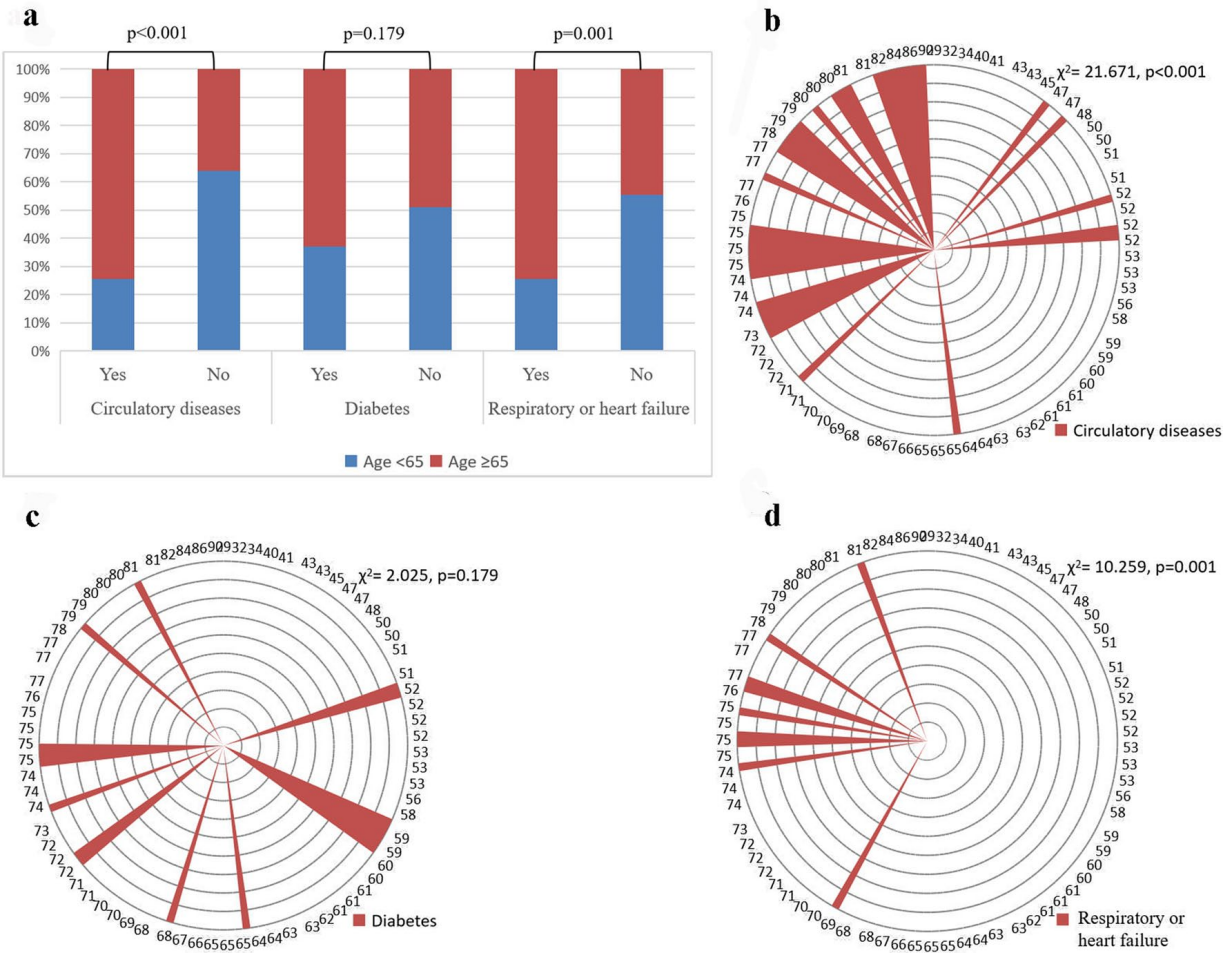
Relationship between concomitant diseases and age. (**a**) Relationship between concomitant diseases and age; (**b**) Relationship between age and diseases of the circulatory system; (**c**) Relationship between age and diabetes; (**d**) Relationship between age and respiratory or heart failure.

As the CEA elevations may be affected by concomitant diseases, further study was conducted on the concomitant diseases of the patients with CEA elevations. The majority of these patients had multiple underlying diseases, mainly diseases of the circulatory system (42.28% [63/149]), endocrine diseases (26.85% [40/149]), and respiratory or heart failure (24.16% [36/149]. In endocrine diseases, 87.5% (35/40) of patients had diabetes. Diseases of the circulatory system and respiratory or heart failure were highly correlated with age (χ^2^ **= **21.671, p < 0.001; χ^2^** = **10.259, p = 0.001) while diabetes was not correlated with age. Immune-related diseases and diseases of the digestive system were less common in BLD (Figs. 2 and 3).

**Figure 4:**
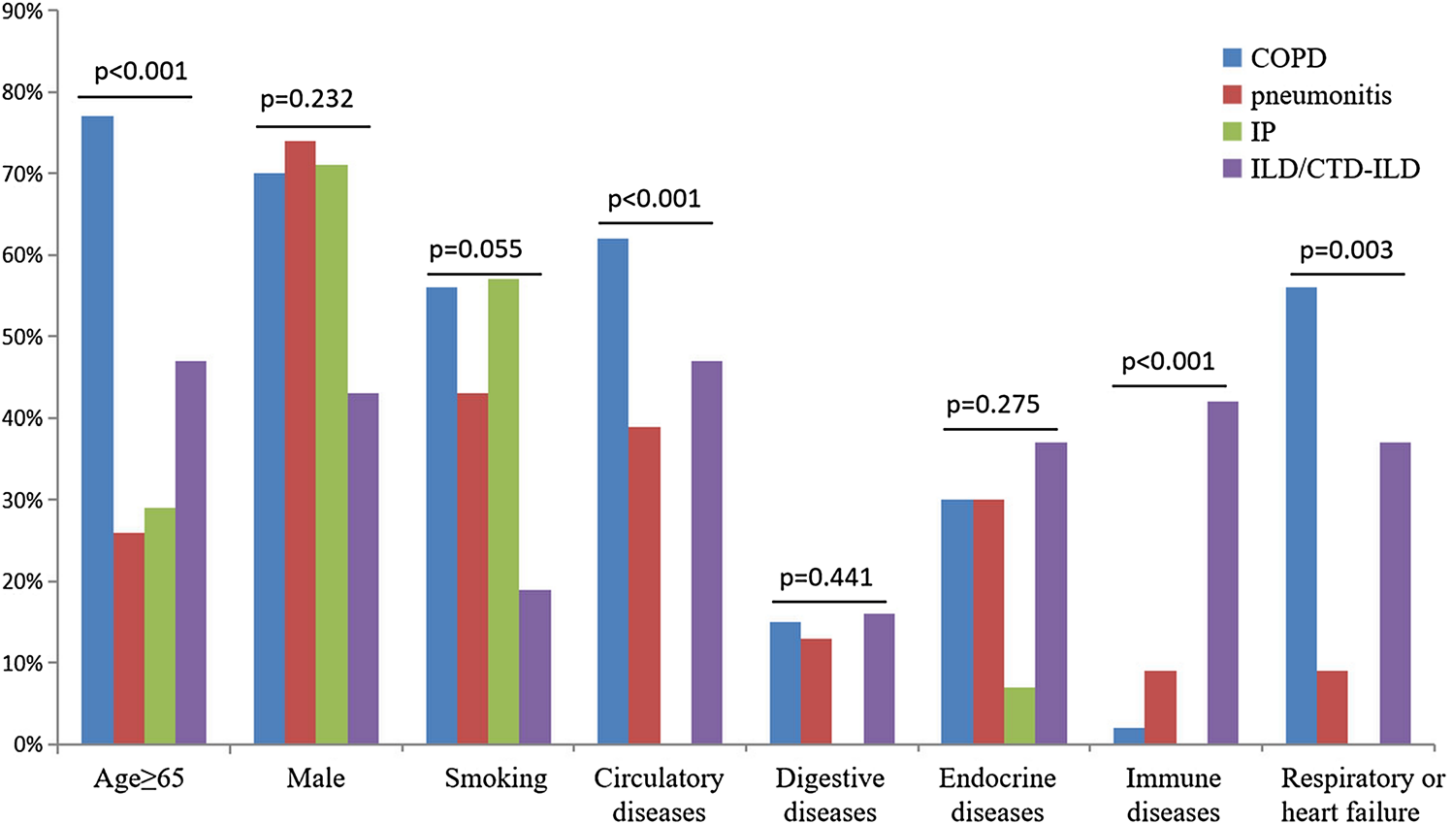
Characteristics of CEA elevations in four types of benign lung disease. *COPD* Chronic obstructive pulmonary disease, *IP* Inflammatory pseudotumor, *CTD-ILD* Connective tissue disease-associated interstitial lung disease, *ILD* Interstitial lung disease.

Among patients with COPD, pneumonitis, inflammatory pseudotumor and interstitial lung disease, which had more than 10 cases with CEA elevations, univariate analysis of variance showed no statistical difference in CEA levels (F = 0.925, p = 0.426). Age and comorbid diseases of the circulatory system, immune disease and respiratory or cardiac failure were significantly different among the four groups (χ^2^ **= **24.212, p < 0.001; χ^2^ **= **18.648, p < 0.001; χ^2^ **= **2 8.638, p < 0.001; χ^2^ **= **14.261, p = 0.003). Sex, smoking status, and endocrine diseases including diabetes (χ^2^ **= **2.897, p = 0.408) and diseases of the digestive system showed no statistical difference among the four groups. COPD patients were older (median age = 74 years, ≥ 65 years = 77%) and had a higher rate of diseases of the circulatory system (62.30%) and cardiorespiratory failure (39.34%). ILD patients had a higher proportion of comorbid immune disease (42.11%) and cardiorespiratory failure (36.84%). Inflammatory pseudotumor patients had few comorbidities (Fig. 4).

### CEA levels of benign lung diseases in the literature

Our PubMed search found 9 eligible studies from 4191 papers^10–18^, including 885 cases of BLD. The sample size varied between 10 and 318. The CEA cutoff value ranged between 4 ng/mL and 12 ng/mL in these studies and the positive rate between 0 and 51%. Different studies included different types of benign diseases, but were dominated by pulmonary tuberculosis, COPD, pneumonitis and benign lung tumors in these studies. There were 53 positive cases, and a mean positive rate of 5.99%. Only two cases had CEA above 20 ng/mL (Table 3).

**Table 3:** Summary of CEA levels in benign lung diseases reported in the literature.

Author	N	Cutoff (ng/mL)	N of CEA > cutoff	Maximum value	Type of disease
Schneider et al.^10^	106	6.4	0	6.4	Pneumonitis and COPD
Sharma et al.^11^	18	5	1 (5.56%)	6.8	Tuberculosis
Wang et al.^12^	87	9.8	2 (2.3%)	10.8	Tuberculosis; pulmonary infection; COPD
Kulpa et al.^13^	96	4	5 (5.2%)	9.5	Sarcoidosis; tuberculosis; fibrosis, asthma; hamartoma; noncancerous tumors; pneumonitis
Nisman et al.^14^	85	5	6 (7.05%)	10^a^	Infectious lung diseases; diffuse noninfectious lung diseases; COPD
Song et al.^15^	124	5	7 (5.65%)	8.93	Tuberculosis; inflammatory pseudotumor; other benign tumors
Beržinec et al.^16^	10	4.61	1 (10%)	6.37	Benign respiratory tract diseases
Gruber et al.^17^	318	12	10 (3.14%)	20^a^	Tuberculosis; pneumonitis; pleural effusion; sarcoidosis; fibrosis; COPD; Benign lung tumors; others
Fahim et al.^18^	41	5	21 (51%)	26^a^	Idiopathic pulmonary fibrosis
Total	885		53 (5.99%)		

*COPD* Chronic obstructive pulmonary disease.

^a^Approximate value.

## Discussion

CEA is a glycoprotein involved in cell attachment and is produced by intestinal epithelia during fetal development and production is ceased upon birth. CEA, which is detected in very small amounts in the serum in healthy individuals, is used clinically as a serological tumor biomarker, especially colorectal carcinoma, and other cancers that are of adenocarcinoma type including gastric cancer and lung cancer. In addition, CEA levels are also elevated in certain benign diseases, including nonulcerative colitis, pancreatitis, liver cirrhosis, ascites, metabolic abnormalities (diabetes, hypothyroidism etc.), peptic ulcer and pulmonary fibrosis^19^. Furthermore, smoking and advanced age also lead to CEA elevations^20^. Hao et al*.*^21^ examined CEA in 70,993 patients with 49 types of diseases and found that the median CEA value was significantly different in 42 types of diseases *versus* healthy subjects, among them, in the descending order, pulmonary fibrosis, pancreatic cancer, uremia, COPD, colorectal carcinoma, Alzheimer’s disease and lung cancer. The study indicated that pulmonary fibrosis (median 4.2 ng/ mL) and COPD (median 3.0 ng/mL) could easily lead to CEA elevations, which in some cases could exceed the median CEA of certain tumors. Literature has shown that 66.1% of IPF patients who were candidates for lung transplant had CEA elevations (cutoff 4.05 ng/mL)^8^. It has been shown that CEA elevation is closely related with severity of lung diseases^8^. Consistently, our current study showed that a greater proportion of patients with COPD and ILD had elevated CEA levels than patients with other types of BLD, and a higher proportion of patients with elevated CEA levels had cardiopulmonary failure. The current study also revealed that very few pulmonary tuberculosis patients had CEA elevations while pulmonary alveolar proteinosis had the highest positive rate among BLD. In addition, COPD patients were older and a higher rate of diseases of the circulatory system and cardiorespiratory failure, which may at least partially explain its relatively high CEA positive rate. Moreover, this study found that 23.48% patients with elevated CEA levels had diabetes. Diabetes exhibits no difference among different lung diseases and didn’t correlate with age of patients, indicating that diabetes might be a very important risk of CEA elevation. However, diabetes itself related to many BLD, such as IPF and COPD, which have been reported by literature^22^. Only 3.11% of our benign lung disease patients had elevated CEA levels, only 0.58% patients had CEA > 10 ng/mL and only 3 (0.06%) patients had CEA above 20 ng/mL. Limited data from currently available literature in PubMed also indicated that only three patients (including one case reprort^23^) had CEA > 20 ng/mL. Therefore, CEA in patients with BLD infrequently exceed 10 ng/mL and rarely exceeds 20 ng/mL.

Previous studies indicate that smoking is a determinant of CEA levels. Alexander et al.^20^ measured serum CEA levels in 276 healthy volunteers, including 154 smokers and 122 nonsmokers using Hansen-Z-gel technique, and showed the mean CEA level (2.7 ng/mL) in smokers was markedly higher than that of nonsmokers (1.9 ng/mL) (p < 0.001). Fukuda et al.^24^ determined CEA levels in 1341 subjects. In male subjects, the mean CEA level in smokers (3.11 ± 1.8 ng/mL) was significantly higher than that of nonsmokers (2.14 ± 1.8 ng/mL; p < 0.01) while in females, there was no difference in CEA levels between smokers (2.11 ± 0.91 ng/mL) and nonsmokers (1.87 ± 2.13 ng/mL). In the current study, for BLD patients, using 5 ng/mL as the cutoff and chi-square test, we found no effect of smoking on CEA positive rate, indicating that smoking may only have insignificant effect on CEA levels.

The mechanisms behind CEA elevated levels in benign lung disease is poorly understood until now. It has been reported that Kupffer cells and alveolar macrophages can endocytose CEA through their surface receptors^25,26^, and many factors may cause the endocytosis dysfunction of these cells. Tanaka et al.^23^ reported a 44-year-old man with diagnosis of pulmonary alveolar proteinosis. CEA level was markedly elevated in his serum (50.6 ng/mL). It was suggested that type II alveolar epithelial cells were those which produced and secreted CEA into the alveoli, and that the dysfunction of alveolar macrophages was secondary to the overingestion of the proteinaceous material in the alveoli. In addition, it has been reported that alcohol can also damage the endocytosis of CEA by Kupffer cells^27^.

Certainly, this study is a retrospective study and cannot exhaust all the causes in search of determinants of CEA elevations and therefore cannot fully exclude other possible contributors to CEA elevations, which may impact on the accuracy of this study. In addition, though this is a large sample study, some BLD have a small size because of their low prevalence, which may affect the accuracy of the results. Of course, the current study population was from single center in China and the study findings may not be applicable to other ethnicities. More accurate data depends on global prospective multicenter study with a large sample size.

In summary, for lung disease patients, if CEA levels rise, we should carry out comprehensive analysis of types of lung diseases, age of patients, and comorbid illnesses. For lung cancer patients, CEA elevation in the course of treatment should prompt clinicians to consider the extent of CEA elevation, apart from disease progression. Drug-induced (including chemical drugs, targeted drugs, and immunotherapeutic agents) pneumonitis, interstitial lung disease, radiation pneumonitis or pulmonary fibrosis, and concomitant respiratory failure should be excluded. Meanwhile, for patients with confirmed lung occupying lesions, if CEA exceeds 20 ng/mL, malignancy should be highly suspected; this will provide certain evidence for clinical diagnosis when biopsy is not feasible.

## Supplementary Information

Supplementary Information.

## Data Availability

The data presented in this study are available on request from the corresponding author.

## Abbreviations

CEA: Carcinoembryonic antigen
COPD: Chronic obstructive pulmonary disease
ILD: Interstitial lung disease
CTD-ILD: Connective tissue disease-associated interstitial lung disease
PAP: Pulmonary alveolar proteinosis
IP: Inflammatory pseudotumor
COP: Cryptogenic organizing pneumonia
BLD: Benign lung disease
IPF: Idiopathic pulmonary fibrosis
