# Supplementary material for: Serum carcinoembryonic antigen elevation in benign lung diseases
Source: Sci Rep. 2021 Sep 24;11:19044. doi: 10.1038/s41598-021-98513-8 (PMC8463604; doi:10.1038/s41598-021-98513-8)
Supplement: Supplementary file 1 — Supplementary Information. [file 41598_2021_98513_MOESM1_ESM.pdf]

Serum carcinoembryonic antigen elevation in benign lung diseases

Yi Yang*, Mingfang Xu*, Huan Huang*, Xiaolin Jiang, Kan Gong， Yun Liu， Xunjie Kuang, Xueqin Yang

Supplementary

Table 1 Patients' characteristics of CEA value above 5 ng/ml

No.	Sex	Age	CEA (ng/ml)	Respiratory diseases	Circulatory diseases	Digestive diseases	Endocrine diseases	Immune diseases	RF or HF	Smoking index
1	F	79	5.00	Pulmonary abscess	Hypertension		Diabetes			0
2	M	64	5.00	Pneumonitis						0
3	F	65	5.28	IP			Diabetes			0
4	M	59	5.33	TB			Diabetes			0
5	M	61	5.61	IP						600
6	F	51	5.80	COP						0
7	F	70	5.14	Bronchiectasis	CAD, Hypertension					0
8	M	53	5.04	COPD						400
9	M	60	5.11	COPD						1200
10	F	59	5.12	COPD	Hypertension		Diabetes, Low T3 syndrome		RF	800
11	M	74	5.12	COPD						800
12	M	63	5.11	COPD			Diabetes			800
13	M	45	5.13	IP						400
14	F	41	5.15	IP						0
15	M	80	5.22	ILD	Hypertension					0
16	M	34	5.26	IP						400
17	F	81	5.26	COPD						0
18	F	32	5.14	PAP					RF	0
19	M	29	5.77	PAP						280

20	M	78	5.22	COPD	CAD, AF, Hypertension				HF	1000
21	F	52	5.23	CTD-ILD	Pericardial effusion			Sjogren's syndrome	RF, HF	0
22	M	72	5.27	TB, Septicemia		Gastric ulcer				0
23	F	88	5.30	COPD	CAD, Pacemaker implantation	Cholecystectomy			HF	0
24	F	63	5.30	CTD-ILD	Hypertension			Systemic lupus erythematosus		0
25	M	76	5.32	COPD						1000
26	M	60	5.36	Pneumonitis	Valvular disease	Cholelithiasis	Diabetes			900
27	M	77	5.37	Pneumothorax	CAD, Hypertension		Hyperthyroidism		RF ,HF	300
28	F	62	5.37	Pneumonitis				AIDS		0
29	F	81	5.39	COPD	CAD, Hypertension		Diabetes			0
30	M	80	5.42	COPD	Hypertension				RF	750
31	M	47	5.43	Pneumonitis	Hypertension					400
32	M	63	5.45	Bronchiectasis						1000
33	F	79	5.45	COPD, PE	CAD, Hypertension				HF	0
34	M	46	5.48	Pneumonitis	Hypertension	Cholecystectomy				600
35	M	75	5.56	COPD	CAD, Hypertension				HF	0
36	F	82	5.58	COPD	Hypertension				HF	0
37	F	56	5.60	Pneumonitis			Diabetes		RF	0
38	M	47	5.43	CTD-ILD	Hypertension			IIM		400

39	M	90	5.71	COPD	Hypertension					0
40	M	77	5.72	COPD	Valvular disease	Gastric ulcer			HF	0
41	M	82	5.72	COPD	Hypertension	Gastric ulcer				800
42	M	62	5.73	COPD				RA		800
43	F	65	5.74	COPD	CAD, Hypertension		Diabetes		RF, HF	0
44	M	68	5.76	Bronchiectasis	Hypertension		Diabetes			0
45	M	53	5.76	Pneumonitis, PE						0
46	F	40	5.78	CTD-ILD						0
47	M	49	5.78	Pneumonitis						600
48	F	52	5.83	TB	Hypertension					0
49	M	43	5.85	Pneumonitis						100
50	F	74	5.97	CTD-ILD		Cirrhosis		RA	RF	0
51	F	55	5.99	PAP	Hypertension					0
52	M	44	6.06	Pneumonitis,					RF	200
53	M	86	6.12	COPD	CAD, Valvular disease					1000
54	M	61	6.16	COPD					RF	0
55	M	52	6.18	ILD			Diabetes			0
56	F	52	6.27	ILD			Diabetes		RF	0
57	M	65	6.29	COPD	Hypertension					400
58	F	65	6.30	Pneumonitis						0
59	M	71	6.37	COPD						0
60	F	61	6.44	TB	CAD		Diabetes			0
61	M	39	6.50	Asthma						0
62	F	69	6.51	COPD	CAD				RF, HF	0

63	F	65	6.50	CTD-ILD	Valvular disease					400
64	F	74	6.65	CTD-ILD	CAD		Diabetes			0
65	M	81	6.55	COPD						1200
66	M	50	6.61	Pneumothorax						0
67	M	75	6.07	IP						600
68	M	34	6.18	IP						0
69	M	50	6.57	IP						600
70	F	65	6.63	COPD						0
71	M	65	6.70	IP						600
72	F	71	6.82	Pulmonary embolism, PE	CAD				HF	0
73	M	70	6.85	COPD		Cholelithiasis，Cholecystectomy	Diabetes			1000
74	M	71	6.98	COPD	CAD					1000
75	M	61	6.25	COP						900
76	M	29	6.26	Pulmonary abscess				AIDS		200
77	F	77	6.35	IP						0
78	M	50	6.61	Pneumothorax						0
79	M	59	7.06	COPD			Diabetes			0
80	F	80	7.09	COPD	Hypertension，CAD	Cholecystectomy	Diabetes			0
81	F	79	7.09	COPD						600
82	M	61	7.09	Bronchiectasis						1500
83	M	75	7.09	Pneumonitis	CAD, Hypertension		Diabetes		RF	800
84	F	72	7.14	COPD	Hypertension				HF	0

85	M	58	7.15	COPD						0
86	M	84	7.15	COPD	CAD, Hypertension				HF	800
87	M	72	7.19	COPD			Diabetes			1040
88	F	79	5.60	Pneumonitis	CAD		Diabetes		HF	0
89	M	77	7.29	Pneumonitis						0
90	M	64	7.31	COPD						400
91	M	63	7.31	ILD						0
92	M	64	7.32	COPD			Diabetes			400
93	M	75	7.37	COPD	Hypertension		Diabetes		RF	200
94	F	68	7.42	COPD					RF	0
95	M	52	7.55	Pneumonitis	Hypertension		Diabetes			0
96	M	52	7.64	COPD	Hypertension		Hyperthyroidism			200
97	M	68	7.74	COPD	Hypertension					400
98	F	48	7.20	Asthma						0
99	M	75	7.37	COPD	Hypertension		Diabetes		RF	1000
100	M	66	7.75	TB			Diabetes			1200
101	F	72	7.90	CTD-ILD	Hypertension		Diabetes			0
102	F	61	8.08	Asthma	Hypertension					0
103	M	78	8.41	COPD	CAD		Diabetes		HF	900
104	M	59	8.56	ILD		Liver transplantation	Diabetes			300
105	M	75	8.95	COPD	CAD					1000
106	M	73	8.12	Pneumonitis	Hypertension	Cholecystectomy	Hyperthyroidism			0
107	M	52	8.18	Pneumonitis	Hypertension					0
108	M	68	8.40	Pulmonary abscess			Diabetes			800

109	M	72	8.45	CTD-ILD	Myocardial infarction, Pericardial effusion			RA, IIM		0
110	M	60	8.70	Bronchiectasis						600
111	M	51	8.56	Pneumonitis				AIDS		300
112	F	80	8.95	COPD	Valvular disease					0
113	M	51	8.98	CTD-ILD			Autoimmune thyroiditis	Autoimmune thyroiditis	RF	0
114	M	80	9.00	COPD, PE					RF	500
115	M	67	9.20	COPD						0
116	M	53	9.43	IP						400
117	M	43	9.43	IP						0
118	M	53	9.43	IP						400
119	F	83	9.57	COPD	CAD	Cirrhosis	Diabetes			0
120	M	68	9.69	COPD						800
121	M	61	9.89	TB						1200
122	M	53	10.01	COPD	Hypertension	Intestinal polyps				500
123	F	43	10.04	Asthma						0
124	M	74	10.08	Pneumonitis	Hypertension		Diabetes			0
125	M	77	10.08	ILD						0
126	M	72	10.18	TB						1200
127	M	77	10.42	Asthma					RF	800
128	M	72	10.75	COPD			Diabetes			500
129	M	85	10.93	COPD	CAD	Cholelithiasis				600
130	M	40	10.95	PAP						200

131	F	47	11.14	COPD						0
132	M	74	11.21	COPD	Hypertension	Cholecystectomy				800
133	F	57	11.22	IP						0
134	M	75	11.26	COPD	CAD		Diabetes		RF, HF	600
135	M	66	11.38	PAP		Gastric ulcer				0
136	M	51	11.73	Pulmonary abscess						400
137	M	78	11.78	COPD	CAD, Hypertension					0
138	M	69	11.78	Pulmonary embolism					RF	300
139	M	74	11.82	COPD	CAD, Hypertension				HF	0
140	M	47	11.97	COPD					RF	600
141	F	81	12.49	COPD	CAD, Hypertension		Diabetes		HF	0
142	M	71	14.35	COPD,	AF		Thyroid adenoma		RF	0
143	M	53	15.13	Pneumonitis, PE						300
144	F	75	17.33	COPD	CAD, Hypertension				HF	0
145	M	70	19.02	CTD-ILD				RA		0
146	F	60	19.23	CTD-ILD	CAD		Diabetes	IIM	HF	0
147	F	76	24.25	ILD		Cholelithiasis			RF	0
148	M	43	28.02	PAP						200
149	F	52	40.12	COP						0

COPD: Chronic obstructive pulmonary disease; CAD: coronary artery disease; AF: atrial fibrillation; TB: Tuberculosis; RF: Respiratory failure:; HF: Heart

failure; PAP: Pulmonary alveolar proteinosis; PE: Pleural effusion; IP: Inflammatory pseudotumor; CTD-ILD: Connective tissue disease-associated interstitial lung disease; IIM: idiopathic inflammatory myopathies; RA: Rheumatoid arthritis; ILD: Interstitial lung disease; COP: cryptogenic organizing pneumonia
